# Restricting Unhealthful Food Advertising to Children and the First Amendment

**Published:** 2011-12-15

**Authors:** Lauren M. Rossen

**Affiliations:** Johns Hopkins Bloomberg School of Public Health, Baltimore, Maryland

## To the Editor:

In their recent article, Harris and Graff describe ways that local governments can restrict the marketing of unhealthful food to children, including limiting store displays and banning commercial billboards (1). The authors state that "to avoid potential First Amendment violations, the [policies] should apply to all signs no matter the message and should be based on non-speech-related considerations such as minimizing visual clutter" or "traffic safety or esthetics" (1). However, instead of veiling the true intent of such restrictions with stated rationales of minimizing clutter and preserving esthetics, local governments could make the argument that these policies are a legitimate exercise of their police power and would pass the *Central Hudson* test (2).

The Supreme Court has repeatedly ruled that to be protected under the First Amendment, commercial speech must not be misleading; this is the first stipulation of the *Central Hudson* test, which is typically applied to decide commercial speech cases (2,3). The advertising of unhealthful food to children is inherently misleading because children are unable to distinguish between purely informational and commercial or persuasive speech (4).

The second stipulation of the *Central Hudson* test is that a substantial government interest must be served by restricting commercial speech (2). The state's interest in protecting the public's health (ie, police powers) surely provides a stronger rationale for restricting advertising to children than minimizing store window clutter or preserving esthetics. State and local governments have a substantial interest in, and rationale for, intervening to address childhood obesity (5).

The third stipulation is that the regulation of commercial speech has to directly advance the government's interest. Substantial evidence document that children are exposed to a tremendous amount of advertising for unhealthful food, and these exposures are associated with negative health effects (4). However, specific local policies to restrict advertising of unhealthful food to children (eg, restricting signage or billboards) need to be evaluated.

The fourth stipulation is that there has to be a reasonable fit between the regulation and the government's objectives. Local policies to restrict unhealthful food advertising to children should be tailored to meet this fourth objective by focusing on child-centered environments (eg, schools, recreation centers). Lessons can be learned from the field of tobacco control. In *Lorillard v. Reilly*, the Supreme Court struck down a restriction in Massachusetts on tobacco advertisements within 1,000 feet of schools and playgrounds because it was unnecessarily expansive and impeded the rights of adults to receive information about a lawful product (3). To meet this fourth objective, local governments must walk the fine line between formulating policies that will be broad enough to be effective but narrow enough to pass muster with the courts. For example, it is unclear how large the buffer around schools or playgrounds should be for restricting signage or billboards for tobacco or unhealthful food.

It is unclear how restrictions on unhealthful food advertising to children would fare if tested in the courts. In reference to restricting food advertising to children, "no court has addressed whether the proposed restrictions would have met constitutional standards. Although the Supreme Court has resisted efforts to curtail commercial speech, if the restriction directly advanced the government's interest in protecting children and was not more extensive than necessary to serve this purpose, commercial speech jurisprudence states that the law would be constitutional" (6).
